# Molecular network analysis for detecting HIV transmission clusters: insights and implications

**DOI:** 10.3389/fpubh.2025.1429464

**Published:** 2025-01-29

**Authors:** Yangyang Liu, Lichun Hua, Wenqian Wu, You Ge, Wei Li, Pingmin Wei

**Affiliations:** ^1^Department of Epidemiology and Health Statistics, School of Public Health, Southeast University, Nanjing, Jiangsu, China; ^2^Department of Ultrasound Diagnostic, Children’s Hospital of Nanjing Medical University, Nanjing, China; ^3^Department of Emergency, Pediatric Intensive Care Unit, Children’ Hospital of Nanjing Medical University, Nanjing, Jiangsu, China; ^4^Department of Clinical Research Center, Children’s Hospital of Nanjing Medical University, Nanjing, China

**Keywords:** HIV-1, phylogeny, molecular network, molecular epidemiology, transmit

## Abstract

**Objective:**

In order to improve knowledge of HIV transmission dynamics and guide preventive and control strategies, this work uses molecular cluster analysis to objectively detect clusters of HIV genetic sequence similarity.

**Methods:**

89 HIV-positive couples provided blood samples, and plasma was separated for pol region gene sequence amplification. Furthermore, analyzed HIV-1 pol fragment sequences from Nanjing patients between 2015 and 2019. HYPHY and Cytoscape were used to generate and illustrate molecular networks.

**Results:**

In this investigation of 89 double-positive pairs, it was discovered that the pairwise gene distance approach properly detected 82.02% of positive couples at an ideal gene distance of 0.014 substitution/loci. With an accuracy of 86.25%, the optimal parameter for the phylogenetic tree and gene distance approach was 90 + 0.045 substitution/loci. A molecular network was built for the Nanjing samples (2015–2019) using the optimum threshold of the previous technique. This network had 487 sequences with one misconnected cluster. There were 565 sequences in the network created by the latter approach that were not incorrectly connected.

**Conclusion:**

For HIV research, molecular cluster analysis provides novel insights. It helps with preventive and control methods by objectively identifying clusters with comparable genetic sequences, which enhances our knowledge of HIV transmission. Further developments will increase its importance for HIV/AIDS research and worldwide prevention.

## 1 Introduction

HIV/AIDS remains a global health challenge, with an estimated 39 million people infected with the virus and 1.3 million new cases globally in 2022 (1). As one of the key players in the fight against HIV/AIDS, the government has responded comprehensively to HIV/AIDS, but HIV/AIDS remains a major public health problem and a major cause of death in China (2). Even though the current prevalence of HIV/AIDS in China is not high, in the more than 30 years since the epidemic in China, there have been several outbreaks (3), and its transmission mode and epidemiological characteristics have undergone great changes (4). In addition, the increasing burden of morbidity and mortality, the emergence of new HIV subtypes/circulating recombinant forms (CRFs) and drug resistance (2), and changing transmission networks are all contributing to the challenges of epidemic prevention and control. Molecular network analysis can identify high-risk groups for HIV transmission and understand transmission patterns, which in turn can help understand the dynamics of virus transmission and optimize the allocation of public health resources (5). and understanding the dynamics of HIV transmission is crucial for effective prevention and control measures.

Traditionally, epidemiological studies have relied heavily on self-reported data from individuals infected with HIV. However, this approach is limited by factors such as recall bias and social desirability, which can lead to inaccuracies in transmission patterns. Molecular network analysis offers a powerful alternative by objectively identifying clusters of genetically similar HIV sequences, providing insights into potential transmission events. A study used tools such as molecular network analysis to identify the variable nature of HIV-1 transmission in MSM to help develop targeted interventions (6). Yan Zhang et al. studied the molecular epidemiological characteristics of HIV-1 by establishing a molecular transmission network, predicting the driving factors of network expansion, and exploring the factors influencing the transmission dynamics (7). The HIV molecular networks reveal associations of direct or indirect transmission, providing a platform for identifying epidemiological characteristics of ^7^ priority areas and high-risk populations for HIV transmission (8). Having implemented multiple HIV interventions in China, the use of molecular networks to track the spread of drug-resistant strains and promote ART is critical to reducing HIV transmission (1, 9).

The HIV-1 molecular network is complementary to the social and sexual network, and essentially uses molecular clusters composed of gene sequences to understand transmission properties (6). HIV molecular clusters, a collection of non-random clusters associated with specific epidemiological patterns among infected individuals, have emerged as a significant tool in HIV research (10). These clusters are characterized by a high degree of similarity among the genetic sequences within them, indicating a close genetic relationship and potential transmission links. The high mutation rate of HIV, which gives rise to genetic variations among infected individuals, makes the identification of these clusters particularly relevant. The smaller the genetic differences between individuals, the stronger the inference of a potential transmission association. By analyzing these clusters, researchers can gain insights into the epidemiological patterns of HIV transmission, identify key populations and factors driving virus spread, and ultimately guide prevention and control efforts (10). Prevention and control cues are obtained through molecular clusters (11–13) of molecular networks, which are able to infer direct or indirect potential risk factors, for a comprehensive picture of transmission (14).

Global HIV/AIDS experts have recognized the value of molecular networks in HIV prevention and control. As the field continues to evolve, the utilization of these networks will become increasingly important in guiding evidence-based interventions and strategies to combat the HIV epidemic. Further developments will increase its importance for HIV/AIDS research and worldwide prevention. In conclusion, HIV molecular clusters and networks still require more precise models to improve the accuracy and representativeness of analytical results (15), and the integration of these tools into HIV research and practice is crucial to guide the global HIV/AIDS response.

In this study, we aimed to analyze the network formation of the four predominant (16) HIV-1 epidemic subtypes (CRF01_AE, CRF07_BC, CRF08_BC, and B subtypes) in China. At the same time these predominant epidemic subtypes are the dominant epidemic subtypes observed in our study samples, consistent with the national distribution of subtype epidemics. Utilizing both the pairwise gene distance method and the phylogenetic tree combined with the gene distance approach, we successfully identified 89 couples with dual positive HIV-1 infections. Our findings provide valuable theoretical insights and technical guidance for refining the construction methodologies and parameter settings of HIV-1 molecular networks tailored for the Chinese context. This research holds significant implications for enhancing our understanding of HIV-1 transmission patterns and facilitating more effective prevention and control strategies in China. We also highlight the challenges and limitations of this approach, offering insights into potential areas for further research and development. By providing a comprehensive overview of the field, we hope to contribute to the ongoing efforts to combat HIV/AIDS globally.

## 2 Methods

### 2.1 Subjects of the study

This study included 89 couples and 1,013 newly diagnosed HIV infections in Nanjing from September 1, 2015 to June 30, 2019. All HIV-infected patients were confirmed by HIV-1 enzyme-linked adsorbent assay and Western blot of Nanjing Center for Disease Control and Prevention. Following the principle of voluntary participation, the informed consent of patients was completed before the implementation of the survey. All couples were identified as intra-couple transmission after epidemiological investigation and HIV-1 subtype identification. Blood samples and general demographic data were collected from these infected individuals.

### 2.2 Gene sequence amplification

Plasma was isolated from blood samples, and HIV-1 RNA was extracted from 200 μL plasma using the QIAmp Viral RNA Mini kit. The HIV-1 pol gene fragments infected by these couples were amplified by RT-PCR and nPCR. RT-PCR Primers: MAW25 (5’-TGGAAATGTGGAAAGGAAGGAC-3′) and RT21 (5’-CTGTATTTCTGCTATTAAGTCTTTTGATGGG-3′). nPCR Primers: PRO-1 (5’-CAGAGCCAACAGCCCCACCA-3′) and RT20 (5’-CTGCCAGTTCTAGCTCTGCTTC-3′). The pol fragment covered 1 ~ 99 amino acids in the protease region and 1 ~ 234 amino acids in the reverse transcriptase region, with a length of 1,060 bp (HXB2: 2253 ~ 3,312). The PCR product was electrophoresed on a 1% agarose gel, and the amplified positive product was purified and sent to a sequencing company for sequencing.

### 2.3 Gene sequence preprocessing and phylogenetic tree construction

The obtained sequences were spliced using the analysis software Chromas. The bases were edited and corrected using BioEdit software. Sequence alignment was performed using MEGA 7.0 software to align sequences. A sequence database of 89 couples and a sequence database of infected persons in Nanjing were constructed. The GTR + I + *γ* model in FastTree software was used to construct a phylogenetic tree by combining all assembly sequences with reference sequences (from the HIV sequence database of Los Alamos National Laboratory, United States), and the Shimodaira-Hasegawa (SH) node value ≥0.70 (16) was used as the basis for clustering. Genotypes were preliminarily determined by clustering sample sequences with international reference strains.

### 2.4 HIV-1 gene dataset in Nanjing City, 2015–2019

The HIV-1 pol gene was successfully amplified in the plasma of 955 infected individuals in Nanjing. Based on this sequence, the HIV-1 gene dataset in Nanjing from 2015 to 2019 was used to verify the parameters obtained by using the sequence of couples to construct the molecular network. The identification of subtypes showed that CRF01_AE and CRF07_BC were the main prevalent subtypes in Nanjing, followed by CRF08_BC and B, and the distribution of the main subtypes in Nanjing was basically consistent with the distribution of subtypes that were prevalent in China.

### 2.5 Construction of molecular networks

#### 2.5.1 Pairwise gene distance method

The paired gene distances between dataset sequences were calculated by the Distance Matrix.bf program using HYPHY 2.2.4 software (17). Meanwhile, Tamura-Nei 93 (TN93) (18) was chosen as the nucleotide substitution model. After calculating the distance between paired genes in HYPHY software, the molecular network was drawn and presented in Cytoscape software. In Cytoscape (13), the number of nodes, links, and networks in a cluster can also be calculated and visualized.

#### 2.5.2 Evolutionary tree joint gene distance method

FastTree3.0 (19) was used to construct an approximately-maximum likelihood phylogenetic tree, and the Shimodaira-Hasegawa-like test embedded in the software was used to calculate the node values of the phylogenetic tree. Cluster Picker (10) was used to extract molecular clusters under different judgment criteria from the phylogenetic tree. The gene distance between pairs of all sequences in each subtree was calculated, and if the gene distance between any two sequences is less than the set threshold (maximum gene distance), the sequences in the subtree form a molecular cluster (20). A maximum likelihood phylogenetic tree was constructed from the fasta format gene sequence using FastTree 3.0 software. Then the corresponding molecular clusters were extracted and the process proceeded. A molecular network was constructed using the extracted phylogenetic tree and gene sequences in the Cluster Picker software and was finally visualized in Cytoscape.

### 2.6 Statistical analysis

SPSS 24.0 was used for data statistical analysis. Categorical data were expressed as percentages (%), and the comparison of rates between different groups was performed using the chi-square test, with a significance level of *α* = 0.05. *p* < 0.05 indicated statistical significance.

## 3 Results

### 3.1 A comparative analysis of two different discrimination methods on the judgment of positive couples

The study included a total of 89 couples with double-positive cases, among which there were 50 pairs of CRF01_AE, 12 pairs of CRF07_BC, and 27 pairs of CRF08_BC. As shown in Table 1, using the pairwise gene distance method with a gene distance set at 0.014 substitutions/site, 73 out of 89 positive couples were correctly identified, resulting in a correct identification rate of 82.02%. At this threshold, 58 clusters were formed, with 52 clusters containing 2 nodes and 6 clusters containing more than 2 nodes. Out of 178 sequences, 153 clustered correctly, yielding a clustering ratio of 85.96%. Consequently, 7 sequences were incorrectly clustered, resulting in a misclustering rate of 4.49%.

**Table 1:** Pairwise gene distance method positive couples transmission association identification.

Gene distance	Clusters (*n*)	Nodes (*n*)	Cluster proportions (%)	Correctly identify couples	Correctness (%)
0.001	9	18	10.11	9	10.11
0.002	21	42	23.60	21	23.60
0.003	28	56	31.46	28	31.46
0.004	34	68	38.20	34	38.20
0.005	41	83	46.63	41	46.07
0.006	47	95	53.37	47	52.81
0.007	55	116	65.17	57	64.04
0.008	57	126	70.79	62	69.66
0.009	58	131	73.60	64	71.91
0.01	59	133	74.72	65	73.03
0.011	61	141	79.21	68	76.40
0.012	63	149	83.71	71	79.78
0.013	61	151	84.83	72	80.90
**0.014**	**58**	**153**	**85.96**	**73**	**82.02**
**0.015**	**53**	**156**	**87.64**	**73**	**82.02**

Note: Bold indicates the most gene-distance or threshold parameters.

Table 2 demonstrates that using the evolutionary tree joint gene distance method with parameters set at 90 + 0.045 substitutions/site, 77 out of 89 positive couples were correctly identified, resulting in a correct identification rate of 86.52%. This method formed 69 clusters, with 66 clusters containing 2 nodes and 3 clusters containing more than 2 nodes. Out of 178 sequences, 154 clustered correctly, with a clustering ratio of 86.52%, and there were no incorrectly clustered sequences.

**Table 2:** Phylogenetic tree combined with genetic distance method for the identification of positive couples with transmission associations.

Node value + gene distance_max_	Clusters (*n*)	Nodes (*n*)	Cluster proportions (%)	Correctly identify couples	Correctness (%)
90 + 0.01	32	64	35.96	32	35.96
90 + 0.015	45	94	52.81	47	52.81
90 + 0.02	55	114	64.04	57	64.04
90 + 0.025	61	128	71.91	64	71.91
90 + 0.03	67	140	78.65	70	78.65
90 + 0.035	71	148	83.15	74	83.15
90 + 0.04	72	150	84.27	75	84.27
**90 + 0.045**	**69**	**154**	**86.52**	**77**	**86.52**
95 + 0.01	30	60	33.71	30	33.71
95 + 0.015	43	90	50.56	45	50.56
95 + 0.02	53	110	61.80	55	61.80
95 + 0.025	58	122	68.54	61	68.54
95 + 0.03	63	132	74.16	66	74.16
95 + 0.035	67	140	78.65	70	78.65
95 + 0.04	68	142	79.78	71	79.78
95 + 0.045	65	146	82.02	73	82.02

Note: Bold indicates the most gene-distance or threshold parameters.

Further comparison between the two methods revealed that with the evolutionary tree joint gene distance method set at 90 + 0.045 substitutions/site, the correct identification rate of positive couples was higher than that of the pairwise gene distance method at a gene distance of 0.014 substitutions/site. Additionally, the misclustering rate of the evolutionary tree joint gene distance method was lower than that of the pairwise gene distance method, and the former method yielded more clusters than the latter.

### 3.2 A molecular network constructed by pairwise gene distance

The pairwise gene distance method (0.014 substitutions/site) was employed to construct the HIV-1 molecular networks in Nanjing City from 2015 to 2019. Among 955 HIV-1 sequences, 487 were included in the network, resulting in a clustering ratio of 50.99%. A total of 82 transmission clusters were formed, including 41 clusters with 2 nodes, 20 clusters with 3 nodes, 8 clusters with 4 nodes, 4 clusters with 5 nodes, 1 cluster with 6 nodes, 1 cluster with 9 nodes, and 7 clusters with 10 or more nodes. The largest cluster contained 119 nodes (CRF07_BC).

**Figure 1:**
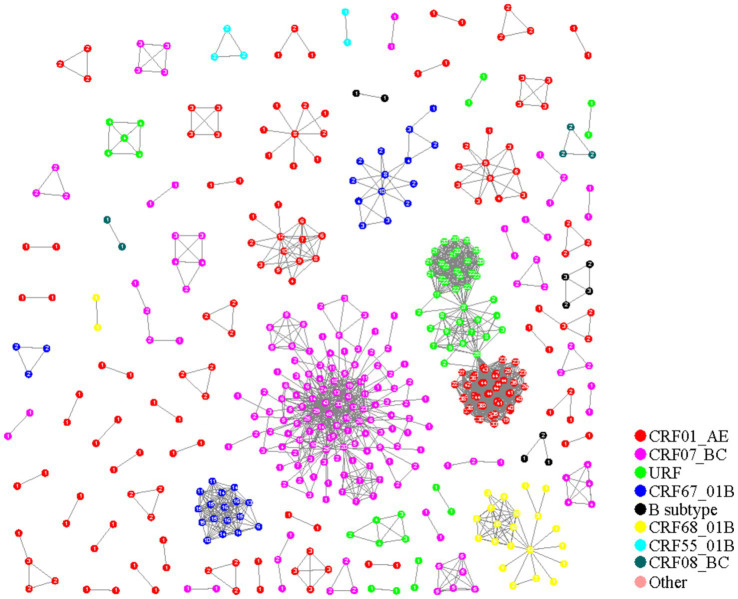
HIV-1 molecular network in Nanjing from 2015 to 2019 (paired gene distance method).

There were 41 molecular clusters of CRF01_AE, consisting of 170 sequences. CRF07_BC had 22 networks with 182 sequences included. CRF08_BC had 2 clusters with 5 sequences included. CRF67_01B had 3 clusters with 34 sequences included, CRF68_01B had 2 clusters with 23 sequences included, CRF55_01B had 2 clusters with 5 sequences included, and B subtype had 3 clusters with 9 sequences included. URF had 8 clusters with 59 sequences included (including 1 cluster erroneously connected with URF and CRF01_AE) (Figure 1).

### 3.3 Molecular network constructed by phylogenetic tree and gene distance method

The systematic evolution combined with gene distance method (90 + 0.045 substitutions/site) was utilized to construct the HIV-1 molecular networks in Nanjing City from 2015 to 2019. Out of 955 HIV-1 sequences, 565 were included in the network, resulting in a clustering ratio of 59.16%. A total of 124 transmission clusters were formed, including 59 clusters with 2 nodes, 32 clusters with 3 nodes, 6 clusters with 4 nodes, 8 clusters with 5 nodes, 3 clusters with 6 nodes, 2 clusters with 7 nodes, 2 clusters with 8 nodes, 4 clusters with 9 nodes, and 8 clusters with 10 or more nodes.

**Figure 2:**
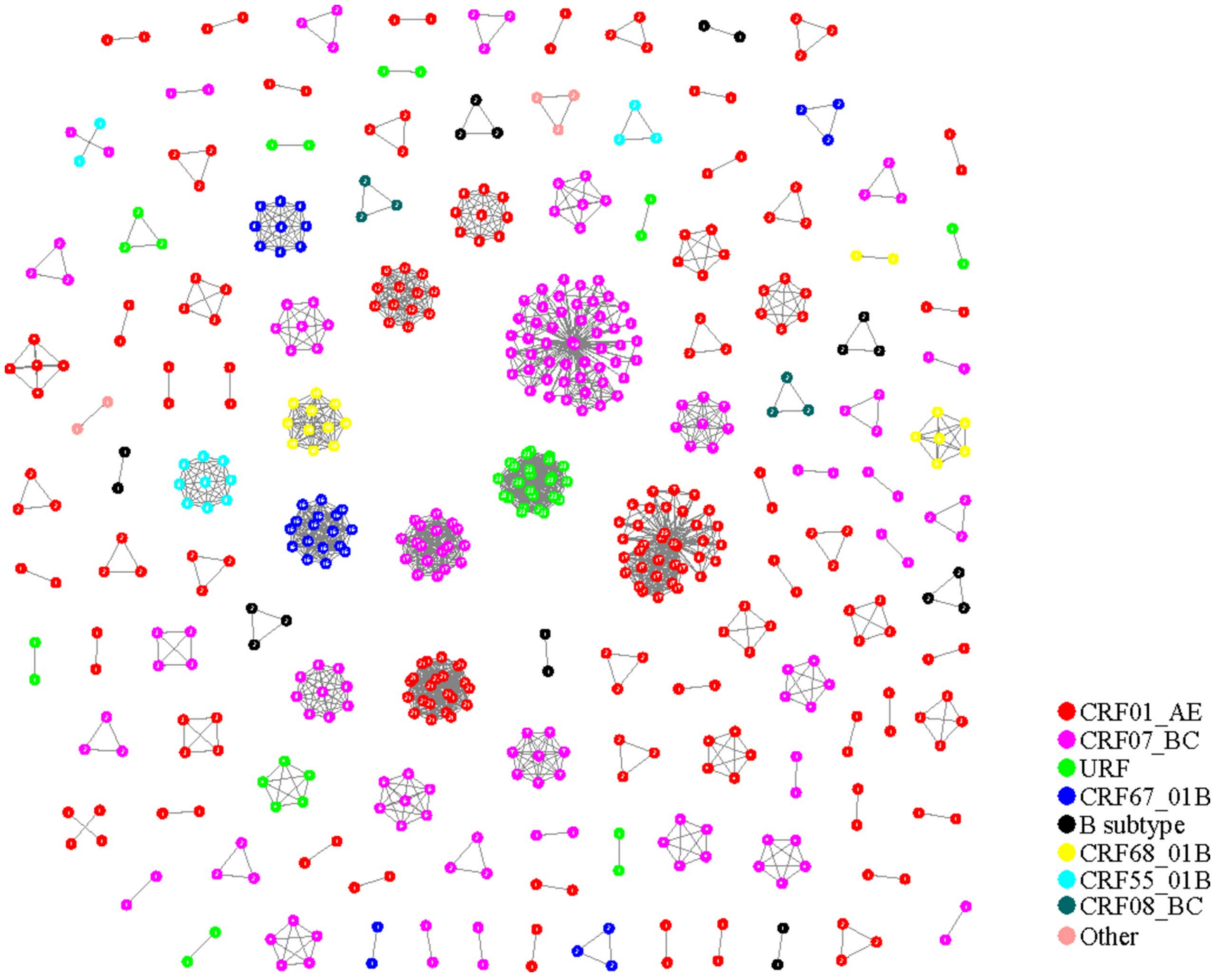
HIV-1 molecular network in Nanjing from 2015 to 2019 (phylogenetic tree combined with gene distance method).

There were 57 molecular clusters of CRF01_AE, consisting of 223 sequences. CRF07_BC had 34 clusters with 188 sequences included. CRF08_BC had 2 clusters with 6 sequences included. CRF67_01B had 5 clusters with 34 sequences included, CRF68_01B had 3 clusters with 19 sequences included, CRF55_01B had 3 clusters with 14 sequences included, and B subtype had 8 clusters with 20 sequences included. URF had 10 clusters with 46 sequences included. Additionally, 5 sequences from other subtypes (2 CRF5801_B and 3 CRF5901_B) formed 2 molecular clusters (Figure 2).

### 3.4 Comparison of the molecular network composition constructed by the two methods

From Table 3, it is evident that the molecular networks constructed using the two methods in Nanjing City from 2015 to 2019 are generally consistent in composition, with networks predominantly consisting of 2 nodes. However, the clustering ratio of the molecular networks constructed using the pairwise gene distance method is higher than that using the evolutionary tree joint gene distance method, and this difference is statistically significant (*χ*^2 = 12.55, *p* < 0.001). The proportion of CRF08_BC subtype is highest in the molecular networks constructed using the pairwise gene distance method, whereas the networks constructed using the evolutionary tree joint gene distance method have the highest proportion of CRF01_AE subtype, and this difference is statistically significant. Furthermore, from Figures 1, 2, it can be observed that the networks constructed using the pairwise gene distance method exhibit potential erroneous transmission associations between URF and CRF01_AE different subtypes, while the networks constructed using the evolutionary tree combined with gene distance method do not show such potential erroneous transmission associations between different subtypes.

**Table 3:** Comparison of the composition of the HIV-1 molecular network between the two methods in Nanjing from 2015 to 2019.

Classify	Pairwise gene spacing method		Evolutionary tree combined with gene distance method	*χ* ^2^	*p*
Network composition	2	41	59	0.58	0.748
	3–9	34	57		
	10	7	8		
Into clusters	Yes	487	565	12.55	<0.001
	No	468	390		
Genotype	CRF01_AE	170	233	258.93	<0.001
	CRF07_BC	182	188		
	URF	59	46		
	CRF67_01B	34	34		
	CRF68_01B	23	19		
	CRF55_01B	5	14		
	CRF08_BC	5	6		
	Other	9	25		

## 4 Discussion

The identification of HIV-1 molecular clusters is essential for understanding the pattern of viral transmission and developing strategies for prevention and control. In this study, we compared the application of pairwise gene distance and phylogenetic tree combined gene spacing in the identification of HIV-1 molecular clusters, aiming to provide a basis for more accurate identification of transmission pairs and construction of potential transmission networks. The choice of genetic distance threshold is crucial. If not chosen correctly, it can lead to many wrong links in the inferred network, making the results difficult to interpret.

The pairwise gene distance method determines molecular clusters based on the genetic distance between sequences (21). For the B subtype, the threshold of this method has varied from 0.005 to 0.015 substitutions/site in previous studies (22), but a broader threshold of 0.045 (23) has been used in other studies. Empirical studies by the CDC have shown that at a pairwise gene distance of 0.0175 substitutions/site, it can effectively differentiate between sequences of positive infection pairs and random sample sequences. The Centers for Disease Control and Prevention recommends a cut-off value of 0.015 substitutions/site for B subtype sequences in its “Identification, Investigation, and Response to HIV-1 Transmission Clusters” guideline (24). Evolutionary tree joint gene distance method is the two most commonly used methods for determining HIV-1 molecular clusters (21). In this study the samples were mainly CRF01_AE, CRF07_BC, and CRF08_BC strains, which are also the predominant subtypes of the current epidemic situation in China (25). Therefore, the use of Nanjing samples in this study is reasonable and applicable. When the threshold was set to 0.014 substitutions/site, the correct identification rate of these positive couples with China’s predominant strains was only 82.02%. It is suggested that for the predominant strains in China, the genetic threshold of the pairwise gene distance method should be appropriately relaxed.

Due to the lack of empirical investigations into positive infection pairs of different HIV-1 subtypes in China, this study first attempted to observe the clustering of major subtype genetic sequences (CRF01_AE, CRF07_BC, CRF08_BC, and B) in public databases using cluster number and clustering ratio as criteria. The cluster number refers to the number of clusters in the same sequence set analysis, and an increased number of clusters is conducive to a detailed analysis of cluster characteristics, thus more accurately identifying potential transmission-related driving factors or targeting active molecular clusters. This study showed that, based on the pairwise gene distance method, the highest number of clusters for the B subtype occurred at a genetic threshold of <0.015 substitutions/site, although this is within the recommended range of 0.005–0.015 substitutions/site. The differences may be related to the sampling depth, geographical and temporal representativeness of the sample sequences. Comparison of the results of the analysis of different subtype sequences showed that the genetic thresholds were different when the cluster number was at its highest, suggesting that different genetic distance standards should be selected for different subtype HIV-1 sequence sets. In contrast, the results of the evolutionary tree joint gene distance method seem more robust, with the maximum number of clusters for all four subtypes occurring at the same threshold of 90 + 0.035 substitutions/site. For the evolutionary tree joint gene distance method, it is more common to report CRF01_AE strains, and the selection criteria are mostly between 90 + 0.035 substitutions/site (26).

When we used two different methods to construct the molecular networks for 89 pairs of HIV-1 infected spouses, the multi-subtypes network was directly constructed instead of constructing networks for different subtypes separately, considering the small sample size. The results showed that when the genetic threshold was 0.014 substitutions/site, the correct identification rate of HIV-1-infected spouses by the pairwise gene distance method was lower than that of the evolutionary tree joint gene distance method (90 + 0.045 substitutions/site), and the pairwise gene distance method had false identification at this threshold, while the evolutionary tree joint gene distance method did not have false identification. When we used two methods and thresholds to construct a molecular networks for Nanjing data from 2015 to 2019, the clustering ratio of the pairwise gene distance method was lower than that of the evolutionary tree joint gene distance method, resulting in a large molecular network and false connections between different HIV-1 subtypes. The difference between HIV-1 quasispecies and founder strains is manifested by the gradual accumulation of genetic distance, and the clusters identified by the pairwise gene distance method are often larger, making it difficult to analyze cluster characteristics in detail. That is, if the genetic variation in the population is smaller, then the genetic distance between individuals will be shorter, which may result in more individuals being grouped into the same cluster (27).

The applicability and accuracy of the pairwise gene spacing method and the phylogenetic tree combined gene spacing method in the case of multi-subtype mixing still need to be further clarified by national sequences. Especially when dealing with complex multi-subtype HIV-1 infection data, these methods may not adequately capture subtle differences between different subtypes, affecting the accuracy of results and in-depth analysis of different subtype characteristics. More genetic sequences can accurately reflect the actual pattern of HIV transmission (28). Since only 89 HIV-1-infected couples were involved in this study, the sample size was relatively small, which limited the ability to directly construct multi-subtype networks. It may be more accurate to construct networks for different subtypes separately, but this strategy was not implemented in this study due to the limited sample size. To overcome these limitations, future studies may consider increasing sample size, collecting more genetic sequences, and conducting in-depth investigations in conjunction with HIV public databases to enhance the extrapolation of results. This study did not conduct protein analysis (WB, ELISA) to pair with genetic data. In the future, we will further use this method to identify genes/proteins involved in the identified clusters to validate molecular aspects and gain a more comprehensive understanding of the molecular mechanisms at play.

## 5 Conclusion

Based on the pairwise gene distance method, different genetic thresholds were selected for different subtypes of HIV-1 sequences in China when the cluster number was highest (all <0.015 substitutions/site), suggesting that different genetic distance standards should be selected for different subtype HIV-1 sequence sets. In contrast, the results of the evolutionary tree joint gene distance method seem more robust, with the same threshold selected for all four main subtypes when the cluster number was highest. When using the pairwise gene distance method, the correct identification rate of 89 couples of HIV-1-infected spouses was lower at a genetic threshold of 0.014 substitutions/site compared to the evolutionary tree joint gene distance method (90 + 0.045 substitutions/site), and erroneous identifications occurred with the pairwise gene distance method at the 0.014 substitutions/site threshold, while no erroneous identifications occurred with the evolutionary tree joint gene distance method. When using the two methods and thresholds to construct molecular networks for Nanjing from 2015 to 2019, the number of clusters identified by the pairwise gene distance method was lower than that by the evolutionary tree joint gene distance method, resulting in larger molecular networks with erroneous connections between different HIV-1 subtypes. The evolutionary tree joint gene distance method may be more suitable for constructing molecular networks with multiple subtypes, while the pairwise gene distance method requires different genetic thresholds for different subtypes to construct molecular networks. A single methodological approach is difficult to support increasingly complex and data-intensive research analyses. Using various methods and theories in combination according to data characteristics and research purposes may make the results more reliable.

## Data Availability

The raw data supporting the conclusions of this article will be made available by the authors, without undue reservation.
